# Mapping of digital pedagogies in higher education

**DOI:** 10.1007/s10639-023-11888-1

**Published:** 2023-06-07

**Authors:** Sonia Santoveña-Casal, Susana Regina López

**Affiliations:** 1 School of Education, Universidad Nacional de Educación a Distancia,, Madrid, Spain; 2 General Academic Secretary, Universidad Nacional de San Martín, Buenos Aires, Argentina

**Keywords:** Bibliometric analysis, Digital methodology, Digital pedagogy, Educational technology, Higher education, Networks of scientific output

## Abstract

The purpose of this research is to analyse the evolution and trends of research into digital pedagogy in higher education through the application of bibliometric analysis and systematic review of scientific output. For the bibliometric analysis, the built-in functions of WoS were used, including *Analyze results* and *Citation report.* The VOSviewer software was employed to construct bibliometric maps. The analysis focuses on studies about digitalisation, university education and education quality, three categories that are grouped around digital pedagogies and methodologies. The sample contains 242 scientific publications, including articles (65.7%), publications published in the United States (17.7%) and publications financed by the European Commission (3.71%). Barber, W., and Lewin, C., are the authors with the greatest impact. The scientific output forms three networks: the “social network” (2000–2010), the “digitalisation network” (2011–2015) and the “network of the expansion of digital pedagogy” (2016–2023). The most-mature research (2005–2009) concerns the integration of technologies in education. The research with the greatest impact (2020–2022) looks at digital pedagogy and its implementation during the situation created by COVID-19. This research shows that digital pedagogy has come a long way over the last twenty years, but it is at the same time a topical area today. The paper opens up future paths for research such as the development of more-flexible pedagogies that can be adapted to different pedagogical scenarios.

## Introduction

Information digitalisation and communication are not only transforming the ways we learn and teach but are also requiring us to take a critical look at universities’ role (Littlejohn et al., 2012). In fact, technology and education are considered intrinsically related (Garrison, 1985), as is demonstrated when distance education is defined on the basis of technology use (Keegan, 1983) and when technology and computing devices are regarded as digital pedagogy’s primary medium (Tabesh, 2018).

Concern over digital education is reflected in multiple actions, most notably the Digital Education Action Plan (2021–2027) (European Commission, 2020), which states that cooperation and exchange in digital education need to be strengthened at the EU level through concrete actions to identify good practices and through greater cooperation among all stakeholders.

Working on the basis of a set of papers (Garrison, 1985; García Aretio, 1999; Santoveña-Casal, 2021; Tarasow, 2010), we established five time periods in technology’s influence on education:

1. Early Internet (early 1900s-1980): This period is characterised by the use of media and the integration of telecommunications in education and multimedia distance education (García Aretio, 1999).
2. Internet expansion (1990–2000): Concepts like e-learning and virtual education are born, seemingly replacing the concept of distance education (Tarasow, 2010).
3. Social period (2000–2010). The Web and Web 2.0 or social web are created, ushering in a paradigm shift (Lankshear & Knobel, 2003). E-learning develops in a constructivist framework (Tarasow, 2010). Mobile learning appears in 2008.
4. Digitalisation (2011–2015): Web 3.0, the semantic web, is developed, and digital classrooms and on-line educator training grow. MOOCs begin.
5. Expansion of digital pedagogy (2016–2023): The smarter, more-interactive Web 4.0 is developed (Schwab, 2017). COVID-19 has a considerable impact on higher education (Khodabandelou et al., 2022; Watermeyer et al., 2021), and universities immediately adapt their methodologies accordingly (Anderson, 2020). The result is a significant expansion of digital pedagogy (Anderson, 2020; Khodabandelou et al., 2022) and therefore a rise in the amount of research done in this knowledge area.

Technology use in higher education has been widely studied (Radianti et al., 2020), but the record shows less research into technology’s integration into specific contexts (Smith & Hill, 2019). Studies of distance education have focused mostly on media, without analysing the key concepts of distance education (Holmberg, 1982). As Garrison (1985) says, if we can identify changes in the conceptualisation and delivery of distance education, we might be able to achieve simplicity and gain an understanding of education and media.

Digital pedagogy is frequently defined in terms of education technology (Jones & Williams, 2010). This approach restricts the concept of digital pedagogy to our use of technology, and it runs the risk of allowing technologies to limit pedagogical creativity (Fyfe, 2011). Digital pedagogy is defined in other ways as well: as a means of teaching using digital resources and technologies (Howell & MacMaster, 2022), as a way to improve or change the education experience (Croxall & Koh, 2013) and address the learning process, collaboration and play (Harris, 2012) and as a technique for working and learning with technologies, generating new, flexible, rich, quality learning experiences (Dangwal & Srivastava, 2016). Digital pedagogy is also conceived as a learning paradigm for facilitating students’ knowledge acquisition through active learning, made up of a rich set of digital tools that make personalised learning and interaction possible (Tabesh, 2018).

That is why the research presented in this article is important. This research shows that digital pedagogy at universities is a promising area of research that is revealing great possibilities for the implementation of new pedagogies in higher education. This paper could give rise to several future lines of research, such as investigations of new, innovative models that respond to today’s needs in the flexible digital pedagogical framework at universities, research into how to achieve a flexible, lasting pedagogical renovation and analyses of how European, national and institutional political variables influence the implementation of a stable, consistent digital pedagogy model in universities.

The objective of this article is to analyse the evolution of and trends in research into the area of digital pedagogy in higher education through the application of bibliometric analysis (Wang et al., 2019) and systematic review (Guirao, 2015) of scientific output. The bibliometric analysis focuses on studies about digitalisation, university education and education quality, three categories that are grouped around digital methodologies and pedagogies.

Bibliometric analysis is a supporting tool academics use to locate unattended areas of research and fields of knowledge by means of co-citation or keywords (Khodabandelou et al., 2022).

Bibliometric studies of digital pedagogy have been done from many perspectives. Some take the classic perspective on distance education (Amoozegar et al., 2018). Others examine the quality of distance education (Means et al., 2009). Some investigate e-learning (Cheng et al., 2014; Khodabandelou et al., 2022), online education and digital education (Santoveña-Casal, 2021), digital literacy (Littlejohn et al., 2012; Spante et al., 2018), improved supervision through the use of technology (Maor et al., 2016) or modern scientific publications on the digitalisation of education (Frolova et al., 2020).

This paper ultimately focuses on the following questions:

1. What are the basic characteristics, and how has the field of research evolved?
2. What authors make up the epistemological network?
3. What thematic network has been generated, and what kinds of networks does the scientific output form?
4. What are the fundamental theoretical bases?

## Theoretical context

### Digital pedagogy and distance education

The origin of digital pedagogy is intrinsically related with distance education (Nanjundaswamy et al., 2021) and has been so for almost two centuries (Spector et al., 2008).

The concept of distance education appeared for the first time in 1892, in documents from the University of Wisconsin (Rumble, 1986). Use of the term became widespread in the 1960 and 1970 s, eventually reaching all of Europe (Verduin & Clark, 1991). Its definition and conceptualisation evolved in step with the emerging technological resources and media of the times (Dede, 1996). Distance education started with the first correspondence courses and gradually evolved hand-in-hand with technologies (Moore et al., 2011), which universities seized upon to adapt their teaching resources to digital formats (Nanjundaswamy et al., 2021).

Distance education is a concept that involves analysing a range of methodologies, the institutional model used and the methodological combination of distance learning and in-person learning (García Aretio, 2001). The concept of methodology is understood to mean not just teaching methods, but also research methods, a situation reflected perfectly in the era of Descartes, when the concept was much more of a whole and therefore a specialist in methodology could be a specialist in either of the two areas (Shulman, 1986).

Much later people began to talk about “online education” or “e-learning”, defined as a pedagogical model that uses digital technologies to generate spaces for dialogue and group learning processes, facilitating interactions and interpersonal relations (Caldeiro, 2013).

### Digital pedagogy and higher education

What seems clear is that technology has had a significant impact on higher education (Amoozegar et al., 2018), giving rise to the development of new pedagogies and models (Bozkurt et al., 2015).

The point is not how we use technological resources (Prestridge, 2012; Milton & Vozzo, 2013), but how we can develop new pedagogies inspired by interaction with machines (Morris & Stommel, 2013). This is a developing concept, and, as Croxall and Koh (2013) report, interest in digital pedagogy has risen significantly in recent years.

Digital pedagogy comprises three knowledge areas that together make up technological pedagogical content knowledge (TPCK) (Koehler & Mishra, 2005). The “T” is technology, meaning technological developments, like the Internet, mobile telephones and open resources. The “P” is pedagogy, which covers practices, procedures, techniques and strategies, objectives, student learning and evaluation of the teaching and learning process. The “C” stands for “content”, the subject matter to be taught. Proper integration of these three elements is fundamental, because education quality does not just mean adding a dash of technology to the established mix; it means developing specific competences and sensitivities for the integration of all three areas (Koehler & Mishra, 2005).

The implementation of digital pedagogy is considered not only a social imperative, but a pedagogical imperative as well (Howell & MacMaster, 2022). Attempts to develop a quality pedagogy have proved rife with shortcomings, however, as when pedagogy lacks a critical focus on higher education. One such critical focus has been developed based on critical digital pedagogy (Morris & Stommel, 2013; Stommel, 2014). This focus is the creation of a critical community that suggests diverse focuses. It converts open networked educational environments into rich critical spaces by going beyond the mere stockpiling of educational content (Stommel, 2014). Other failings in the development of digital pedagogies at universities were made painfully evident during the COVID-19 pandemic, revealing the need for a change in the education system (Watermeyer et al., 2021). The pandemic revealed just how vulnerable universities are (Aljanazrah et al., 2022) and how necessary it is for them to implement new, flexible digital methodologies for learning and teaching (Barana et al.,2021). It also became clear that technologies do not necessarily improve learning (Ng’ambi et al., 2016). As Azionya and Nhedzi (2021) assert, a more-inclusive, more-flexible pedagogy is needed that reduces digital and educational inequalities.

In fact, the Multirank report (Roman, 2020) clearly states that “60% of universities reported online learning provisions in their strategic planning pre-COVID-19, but only few appeared to be prepared for a quick shift to full online programs”. What pedagogical changes happened were actually based on an emergency remote teaching model, in a one-off response to a sudden situation (Czerniewicz et al., 2020; Hodges et al., 2020). For this reason, the education alternatives created during COVID-19 cannot be mistaken for long-term solutions and proposals based on the universal design for learning (UDL), i.e., on flexibility and inclusiveness in learning environments, and on student-centric teaching to guarantee access to education for students of all kinds (Hodges et al., 2020).

Flexible pedagogies are based on many things, including the importance of facilitating access and adaptation to students’ preferences (flexibility in terms of pace, place, contents, learning style, assessment, individual or group work) (Ling et al., 2001), flexibility as a means of modifying the current education system and achieving flexible, open, global, innovative education (International Council for Open and Distance Education, 2009) and flexibility and variety of time, contents, requirements, resources and delivery options in education (Collis & Moonen, 2001). Flexible pedagogy has also been seen as the pedagogy that makes learning anytime and anywhere a reality, but this view reduces the term to the concept of ubiquitousness. In short, before a flexible learning model can be created, answers must be found to the questions of *when* and *where* to learn (space and time), *how* to learn (way of learning/teaching), *what* to learn (contents) and *with whom* to learn (network and community) (Collis & Margaryan, 2007; Willems, 2011). So, one fundamental aspect of flexible pedagogy is accessibility and attention to the universal design for learning.

## Method

### Sample

The sample contains 242 publications from the Web of Science (WoS), from 2005 to 2022, in the area of digital pedagogies in higher education, in English and Spanish, from the category *Education Educational Research*, regardless of the database of origin. The search chain is:

TOPIC “Digital technology*” OR “Digitization*” OR “Digitisation*” OR “Digitali*” AND TOPIC “Higher education*” OR “Higher education* instituti*” OR “University” OR “High education*” OR “high education* superior*” OR “Higher education* superior*” OR “College*” OR “Institution* of higher learning*” AND TOPIC “Educational quality” OR “Educational improvement” OR TOPIC “digital methodolog*” OR “virtual methodolog*” OR “digital educational sciences*” OR “digital Pedagog*”

The sample is made up of 159 articles (65.7%), 68 proceeding papers (28.09%) and 16 book Chaps. (6.61%). Twelve early access articles (4.95%), nine review articles (3.71%), six editorial material articles (2.48%) and two book reviews (0.82%) are analysed as well.

### Data analysis

This research uses bibliometric analysis designed for measuring scientific output (Wang et al., 2019) and systematic review (Guirao, 2015) to identify, evaluate and interpret the scientific output (Fink, 1998) on pedagogy and digital methodology in higher education in the WoS Core Collection database over the last 17 years.

The search was run on 10 January 2023. The bibliometric analysis employed the functions built into WoS, including “Analyze results” and “Citation report”. VOSviewer software tools (Van Eck & Waltman, 2010) designed to construct bibliometric maps were also used to provide detailed maps of scientific output and therefore to enable the analysis of similarities, clusters and networks, as well as citations and co-citations used by multiple researchers (Guo et al., 2021; Khodabandelou et al., 2022; Van Eck & Waltman, 2013).

First, the WoS database search was run for digital pedagogies in higher education, using the chain given above.

Next, the references were extracted into plain text files, including all options (author, title, source, abstract and cited references).

Lastly, the plain text files were entered into the VOSviewer software to extract the various networks of scientific output.

**Fig. 1:**
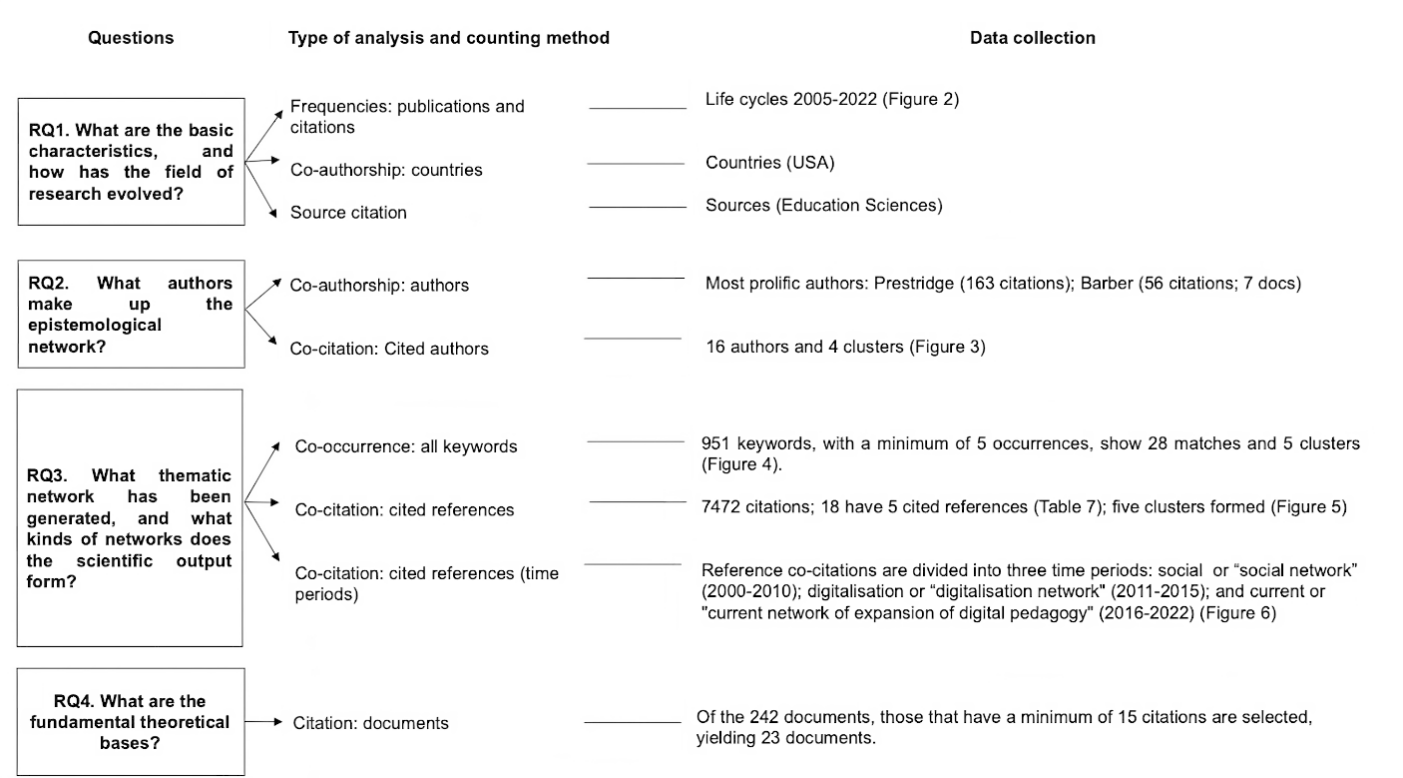
Correspondence between research question, type of analysis and counting method and data collection

The mapping of scientific output in the form of articles, book chapters, proceeding papers, early access articles, review articles, editorial materials and book reviews was done on the basis of a descriptive analysis of the sample (life cycles of the field of study, internalisation and sources), analysis of co-authorship, co-citation of authors and references, keyword co-occurrence and document citation (Fig. 1).

**Fig. 2:**
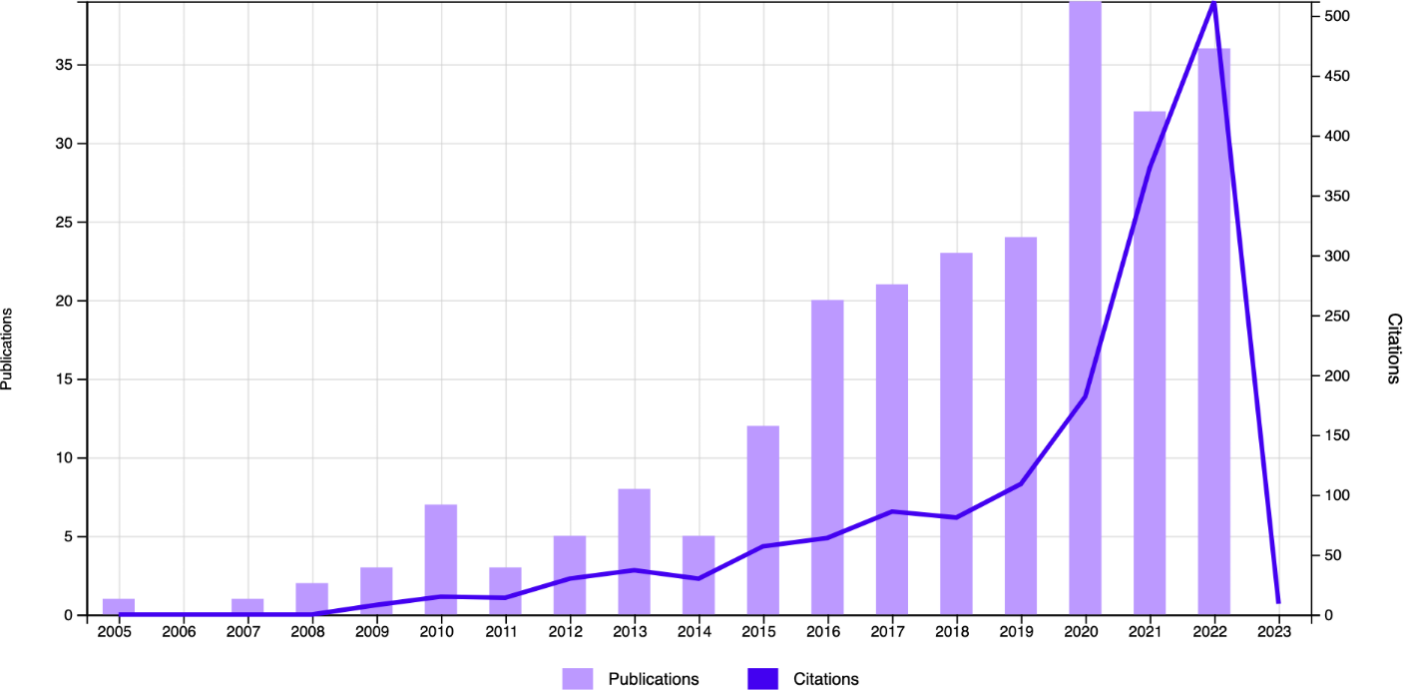
Life cycles of the field of study (frequency)

**Fig. 3:**
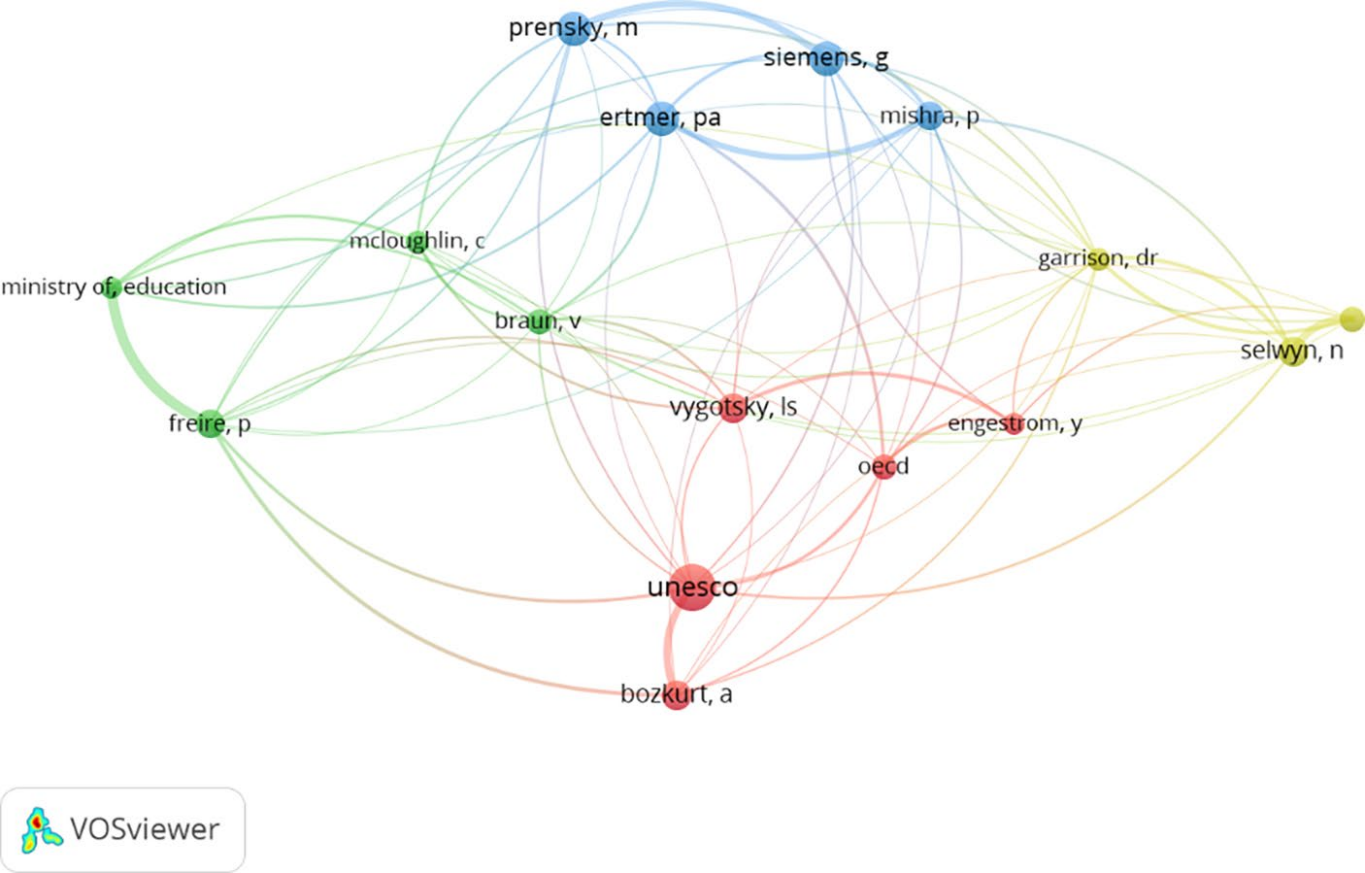
Invisible network of authors (author co-citation)

**Fig. 4:**
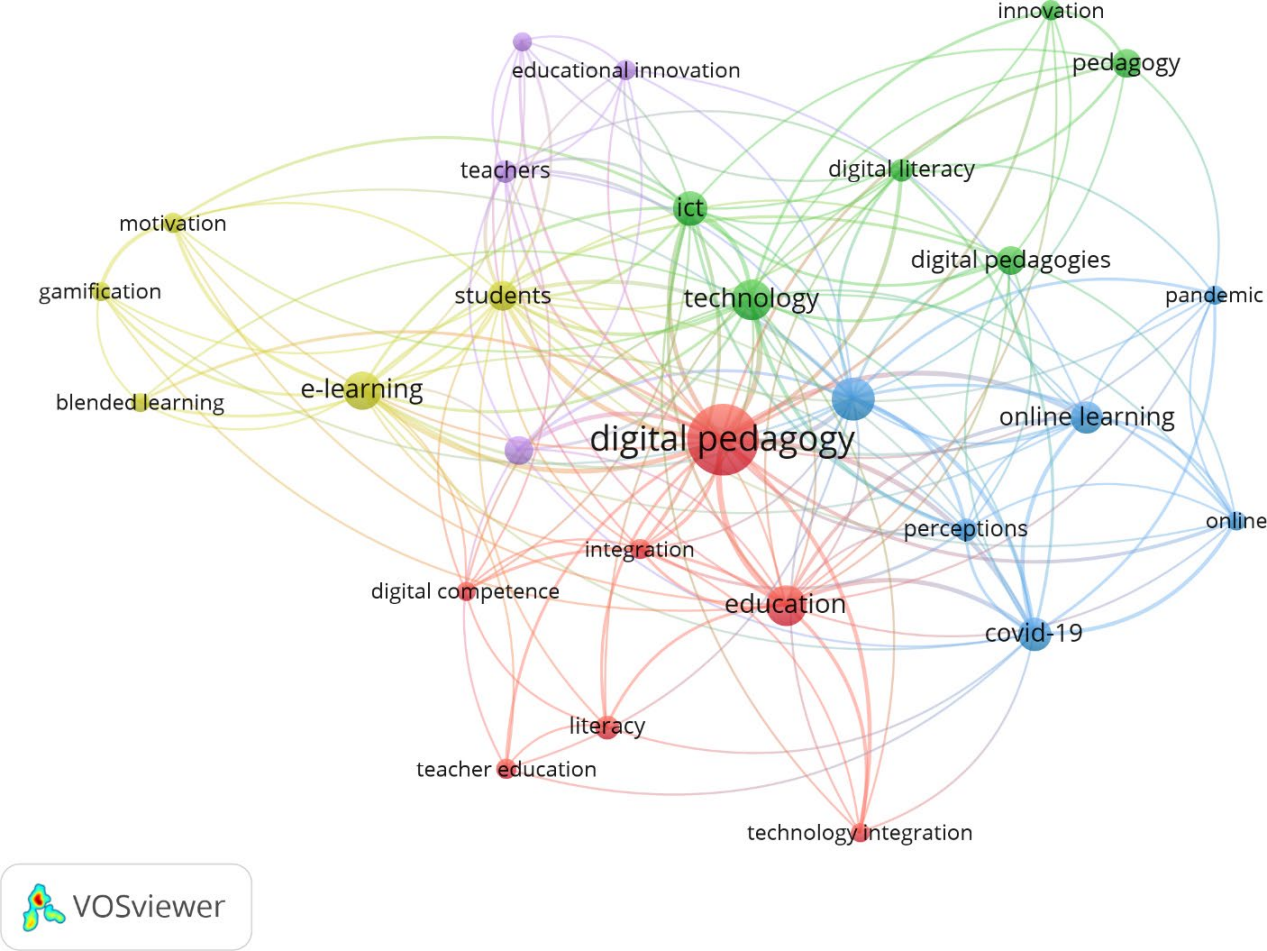
Thematic mapping (term co-occurrence)

**Fig. 5:**
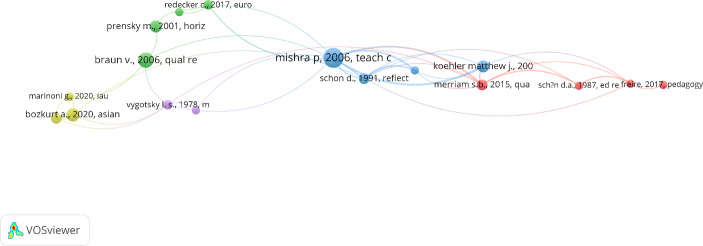
Map of overall scientific output (reference co-citation)

**Fig. 6:**
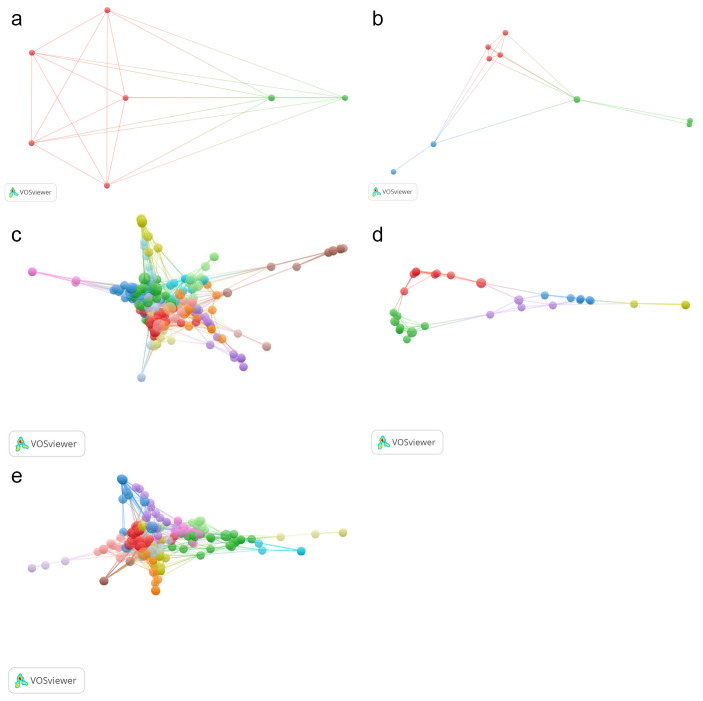
Maps of scientific output by time periods (reference co-citations). **(a)** Social Period (2000 to 2010) – Social Network. Classic documents published from 2005 to 2010 (14 documents, 5.8%) **(b)** Digitalisation Period (2011-2015) – Digitalisation Network. Documents produced in intermediate phases (33 documents, 13.6%) **(c)** Current Period (2016-2022) – Current Network of Expansion of Digital Pedagogy. Current documents (195, 80.57%) **(d)** 2016-2018, 26.44% **(e)** 2019-2022, (131 documents, 54.1%)

The research methodology, presented below, was based on the research questions and the results found (Fig. 1), as follows:

1. Descriptive analysis: trends and evolution of the research field, internalisation and sources.
  An answer was found to question 1, “What are the basic characteristics, and how has the field of research evolved?”
  The following were subjected to descriptive analysis:
  - Publication frequency and citation frequency. The life cycles for 2005 to 2022 were found (Fig. 2).
  - Co-authorship based on countries. The United States is the country with the most publications.
  - Source citations. The most-cited source is *Education Sciences.*
2. The epistemological network of authors.
  An answer was found to question 2, “What authors make up the epistemological network?”
  The following points were analysed:
  - Co-authorship of authors: Analysis of direct relationships through co-authorship, to identify the networks of direct inter-author collaboration. The author with the highest number of citations is Prestridge, S., with 163 citations.
  - Co-citation of cited authors: Analysis of the network of professionals and experts of recognised prestige and expertise in the field of study, by means of indirect relationships. Sixteen authors and four clusters were found (Fig. 3).
3. The thematic networks and networks of scientific output.
  ***The scientific output was divided into three time periods or distinct networks: the social period or “social network”, from 2000 to 2010; the digitalisation period or “digitalisation network”, from 2011 to 2015; and the current period or “current network of expansion of digital pedagogy”, from 2016 to 2022.
  An answer was found to question 3, “What thematic network has been generated, and what kinds of networks does the scientific output form?”
  - 3.1. Co-occurrence of all keywords: A study of term co-occurrence (joint keywords, based on coincidence or concurrence) was conducted, using the terms appearing in the titles of the publications printed during the entire target period. The terms appearing at least five times were selected, yielding 951 keywords and five clusters (Fig. 4).
  - 3.2. Co-citation of cited references: Co-citation of cited references, defined as the number of references that are cited and have two publications in common (Van Eck & Waltman, 2013), was used to analyse the evolution of output, yielding 7472 citations (Table 7) and five clusters (Fig. 5).
  - 3.3. Co-citation of cited references by time period: Reference co-citations were classified into the social time period or “social network” (2000–2010), the digitalisation time period or “digitalisation network” (2011–2015) or the current time period or “current network of expansion of digital pedagogy” (2016–2022) (Fig. 6).
4. Analysis of the fundamental theoretical basis of the knowledge area.
  An answer was found to question 4, “What are the fundamental theoretical bases?”
  The following points were analysed:
  - Citation of documents: The basic publications and theories in the target field were ascertained according to the number of times each document was cited. The most often-cited publications were established as the publications that have contributed to the field. Two hundred and forty-two documents were cited; 23 of them were analysed.

The results are presented in four sections: (1) descriptive analysis: trends and evolution of the research field, internalisation and sources; (2) the epistemological network of authors; (3) the thematic networks and networks of scientific output; (4) analysis of the fundamental theoretical basis of the knowledge area.

### Descriptive analysis

The scientific output concerning digital pedagogy and higher education has certainly evolved over the last 13 years (Fig. 2). The earliest interest in the research area is found between 2005 and 2009, when 2.89% of the scientific production was generated. In 2008 the term “MOOC” appeared for the first time in Canada to describe a set of connectivist experiences in education (Stommel, 2015), as reflected in 2009 with three publications. From 2009 to 2014 there was an unstable period responsible for 13.45% of the scientific output, with two peaks of interest, one in 2010 (3.04%) and one in 2013 (3.48%).

In 2015 scientific interest stabilised. The COVID-19 pandemic in 2019 caused a significant spike in the amount of research published in 2020. In 2021 and 2022 interest in the knowledge area continued. In the last three years (2020, 2021 and 2022), 42.17% of the publications appeared, demonstrating keen interest in the knowledge area during that time.

A gradual increase can also be observed in the number of citations received, peaking in 2022 with 32.55% of all citations. The average number of citations per item is 6.64, with an H-index of 17.

The three countries with the highest scientific output and the highest number of citations received are the United States (17.7%), the United Kingdom (12.39%) and Spain (10.74%). International relations present three groups of interactions (Fig. 3). The main source of funding is the European Commission (N = 9; 3.71%). The most cited source is *Education Sciences* (25.71%).

### Epistemological network of authors

The authors with the highest scientific output are Barber, W. (56 citations and seven documents), associate professor of the School of Education (Ontario Tech Teaching Award), and Lewin, C. (five documents), professor of education at Manchester Metropolitan University. The author with the highest number of citations is Prestridge, S., 163 citations, associate professor, School of Education and Professional Studies, Griffith Institute for Educational Research.

The lead author in co-citations is UNESCO, the most highly cited author in the network (8.63%) and the author with the highest number of connections (6.78%) (Fig. 3).

### Thematic networks and scientific output networks

The centralised network (red cluster) has the highest number of connections (24) and co-occurrences (65) of any of the clusters in Fig. 4, which is connected with the rest of the clusters and has direct relationships with “Education”, “Teacher education”, “Digital competence”, “Technology integration” and “Integration”. The mean publication date is 2018. The blue cluster’s centre is “Higher education”, which is related with topics like “COVID-19”, “Online”, “Online learning”, “Pandemic” and “Perceptions”. Its mean publication date is 2020. “Technology”, the centre of the green cluster, is related with “Pedagogy”, “Innovation”, “ICT”, “Digital pedagogies”, “Digital literacy” and “Technology”. The green cluster’s mean publication date is 2017. “E-learning” stands at the centre of the yellow cluster and has connections with “Gamification”, “Motivation” and “Students”; this cluster has a mean publication date of 2017. Lastly, the purple cluster’s centre is “Educational technology”, which is connected to “Educational innovation”, “Facebook” and “Teachers”. Its mean publication date is 2018.

The reference co-citation analysis (Fig. 5) shows that in the centre of the network (blue cluster) lie the references related with teacher study in higher education, educational technology and/or teaching with technology (Koehler & Mishra, 2009; Schön 1987, 1991), Shulman’s construct (1986) and the three categories of knowledge (subject matter content knowledge, pedagogical content knowledge, curricular knowledge) (Mishra & Koehler, 2006; Shulman, 1986).

This cluster is related with the red cluster through Schön (1987, 1991), a classic text that identifies the most important research areas addressing social interaction in connection with the childhood development of language and cognition. Merriam and Tisdell (2015) present a guidebook for understanding, designing, conducting and presenting qualitative research studies. Littlejohn et al. (2012) study digital literacy in higher education. These documents are related with one of the cornerstones of critical pedagogy, *Pedagogy of the Oppressed* (Freire, 2017), which proposes a new relationship between teacher, student and society.

The green cluster is a cluster of theoretical analyses and benchmark studies on the qualitative analysis method (Braun & Clarke, 2006), the European framework for the development of pedagogical digital competence (Redecker, 2017), a new theoretical construct for learning in the digital era (Siemens, 2004) and the analysis of today’s students (“digital natives”) (Prensky, 2001).

The yellow cluster is made up of research done in 2020 on COVID-19’s impact on education at different levels and in different countries (Bozkurt et al., 2020; Hodges et al., 2020; Marinoni et al., 2020) and universities’ difficulties in implementing distance education (Marinoni et al., 2020). The research distinguishes between planned online learning experiences and learning experiences forced on line during COVID-19 (“emergency remote education”) (Bozkurt et al., 2020; Hodges et al., 2020).

Hodges et al. (2020) present an interesting study on what is called “emergency remote teaching”. They explain that the education alternatives dashed together during the pandemic must not be confused with long-term solutions; long-term plans must include UDL-friendly proposals, and thus must focus on the flexibility and inclusiveness of learning environments. They also point out how the term “online learning” may be politicised and how, although blended learning was readily included on political agendas, its application varied significantly from university to university.

In addition, there are two fundamental texts, Freire (1970) and a selection of essays by Vygotsky (1978), based on the dialectical materialist theory of cognitive development.

Lastly, the reference co-citations were analysed. They were found to fall into three periods and three networks (Fig. 6).

The **social network** is made up of a group of seven publications. All are connected to each other, all have the same importance, and all address similar topics having to do with theoretical analyses and educational technologies: theoretical frameworks (Gallagher, 1992), educational technology (Mishra & Koehler, 2006), meta-learning (Biggs, 1987), maths learning (inter alia, Calder, 2006 et al.; Povey 1997) and cyberspace (Cousin, 2005).

The **digitalisation network** spans 14 productions and four topics. Schön (1987, 1991) lies at the centre, explaining how reflection in action operates in professionals. The network presents a range of methodological focuses in a heavily digitalised context: online communities (Lin & Lee, 2006), teachers integrating technology into their pedagogy (Mishra & Koehler, 2006), the importance of education, technology and innovation in learning (Bolstad et al., 2012), synchronic communication and post-graduate e-learning (Garrison, 1985; Garrison et al., 2000) and Web 2.0 and how it influences learning (Greenhow et al., 2009). There is a second research group that addresses the term “methodology” as research methodology (inter alia, Eisner 1998; Patton, 2002; Merriam & Tisdell, 2015).

Scientific output has increased significantly in recent years, generating a 12-group network with a larger number of relationships. The star-shaped **current network of expansion of digital pedagogy** is the most centralised network and has the greatest weight in the scientific field (a larger number of citations). It has three groups of connections: the benchmark study on the qualitative analysis method (Braun & Clarke, 2006), the analysis of education experiences during the pandemic (Bozkurt et al., 2020) and the proposal of the theory of pedagogical content knowledge (Mishra & Koehler, 2006).

### Fundamental theoretical bases

The references with the highest numbers of citations belong to the social network and the current network of expansion of digital pedagogy.

The most mature scientific output, spanning the last 13 years, belongs to the social network. This output addresses the integration of technologies into higher education, from the perspective of the Social Network 2.0 (Hemmi et al., 2009), integration with maths (Sacristán et al., 2009), the development of the higher education training competences needed in the knowledge society (Clarke & Clarke, 2009) and mobile commerce and digital pedagogies (Luke, 2005). Continuing development is seen in authors’ interest in the influence of digital technologies in this period. Other research looks into digital technologies’ influence on encouragement for learning based on digital problems (Barber et al., 2015) and podcasting for learning in online teacher education (Forbes & Khoo, 2015).

This trend continues over the last two years, forming the current network of expansion of digital pedagogy. Scientific output has analysed the influence of technologies on different methodologies and digital education resources such as OERs (Cooney, 2017), virtual reality as a means of improving university students’ communication skills (McGovern et al., 2020), influence on academic performance at university (Frolova et al., 2020) and digital competence for educators (Cabero-Almenara et al., 2020). The impact of the COVID-19 pandemic is clear in this network; the foremost of the publications (those with the highest numbers of citations) deal with the influence of COVID-19 on digital pedagogies and university teachers (Watermeyer et al., 2021; Giovannella et al., 2020), digital pedagogies and equity (Greenhow et al., 2021), digital pedagogies and teacher training (la Velle et al., 2020) and digital pedagogies and university students’ perception of distance learning (Gonçalves et al., 2020).

## Discussions and conclusions

The conclusion in response to the first research question is that scientific output concerning digital pedagogy and higher education has indeed evolved over the last 13 years. The area has in fact covered a great deal of ground. The European Commission has been the biggest funding source, proof of the Commission’s interest in and concern over the adaptation of pedagogies to more-digital models along the lines of the Digital Education Action Plan (2021–2027) (European Commission, 2020).

Second, the epistemological network of authors is made up primarily of Barber and Lewin. Prestridge is the author with the highest number of citations, and UNESCO is the leading author in co-citations.

The conclusion in response to the third research question is that analysis of the evolution of topics and research clearly reflects the COVID-19 pandemic’s impact on higher education (Khodabandelou et al., 2022; Watermeyer et al., 2021) and the need to adapt universities’ methodology to bring about a significant expansion of digital pedagogy (Anderson, 2020) and therefore a significant increase in the amount of research being done in this knowledge area. The publications with the highest impact in the scientific field and the highest number of citations have to do with the impact of COVID-19 on digital pedagogies (inter alia, Watermeyer et al., 2021). These publications have been released in the last two years.

The scientific output forms three networks, the social network (2000 to 2010), the digitalisation network (2011 to 2015) and the network of expansion of digital pedagogy (2016 to 2023). The most-mature research (publications dating from 2005 to 2009) focused on the integration of social networks in education (the social network). As interest in these topics continued, the digitalisation network (2011 to 2015) was formed, followed by the earliest tendrils of the network of expansion of digital pedagogy (2016 to 2023). By 2017 “technology”, “e-learning” and “educational technology” were the most frequent concepts. Gradually, however, the concept of digital pedagogy became part of the scene (2018), and the current network of expansion of digital pedagogy took firm shape. This network was boosted significantly by the unforeseen COVID-19 situation, which caused the scientific output to increase exponentially (by 54.1%).

Scientific output has been analysed to answer the fourth research question, “What are the fundamental theoretical bases?”

The fundamental theoretical foundations of the knowledge area lie in the social network (connections with the earlier publications from 1987 to 1995, presenting theoretical analyses and research concerning educational technology and learning) and the network of expansion of digital pedagogy (whose papers focus on the COVID-19 pandemic’s influence on the academic community and on digital pedagogies, among other subjects).

Coherent, significant theoretical frameworks are offered, such as Shulman’s model (1986), critical pedagogy (Freire, 2017), dialectical materialist theory (Vygotsky, 1978), frameworks referring to teachers in higher education, educational technology and/or teaching with technology (inter alia, Mishra & Koehler 2006) and more-modern contributions made in the context of the digital society, like the work of Siemens (2004) and Prensky (2001).

The main gap in the research is due to a general lack of planned, long-lasting digital pedagogical proposals flexible enough to respond to the needs of the different universities, curricula and student profiles. The highest-impact publications look at pedagogical changes made during the pandemic and examine the emergency teaching model (Bozkurt et al., 2020; Czerniewicz et al., 2020; Hodges et al., 2020). Multiple studies examine the shortcomings and difficulties of adapting university education to cope with the pandemic (Marinoni et al., 2020), and many authors present pedagogical models (inter alia, Hodges et al., 2020; Morris & Stommel 2013; Stommel, 2014), but no-one offers flexible pedagogy-based alternatives enabling teachers to create new methodologies capable of implementing entire programmes on line instead of forcing educators to cobble together ad-hoc responses to the COVID-19 situation, as indicated by Roman (2020). In fact, most of the target publications do not study flexible pedagogy. There are a few exceptions, such as Hodges et al. (2020), who point out the importance of heeding the universal design for learning. Furthermore, the majority of the studies that do refer to flexibility in learning or in methodology do so in reference to a particular aspect or a specific instance, for example, when discussing the blended model with a flexible structure (Calderón et al., 2020), when emphasising the importance of continuous, specialised, multidisciplinary, flexible military training (Marchisio et al., 2017), when reviewing the possibilities for a more-flexible technology-based system during the pandemic (Aljanazrah et al., 2022) or when explaining the pandemic’s impact on education (Bozkurt et al., 2022).

It is also observed that the research that dwells the longest on how to apply new models to create greater flexibility is not the research with the greatest impact in the sample. There are two exceptions, Hodges et al., (2020), whose work has just been mentioned, and Calder et al. (2021), who explain how the flexibility gained by combining formal and informal digital pedagogies is one advantage of the pedagogical model that came together during the pandemic. The rest of the research focuses on the development and application of multiplatform software as a means of developing digital methodologies that respond to new needs and make the learning process more flexible (Merayo et al., 2015) and how flexible learning is taking digital pedagogies more and more into account (Benade, 2016; Ranga & Etzkowitz, 2013). All these aspects are highly important for the development of a flexible digital pedagogy in the framework of higher education and suggest fundamental points to bear in mind in future research for the study of new, innovative models that can respond to current needs in a flexible digital pedagogical framework for universities.

In short, researchers have definitely identified vulnerabilities in universities (Aljanazrah et al., 2022), shortcomings in the development of university-level digital pedagogies (Roman, 2020; Watermeyer et al., 2021) and the need to roll out new, flexible digital methodologies for learning and teaching (Azionya & Nhedzi, 2021; Barana et al., 2021), and yet universities do not seem to have made any permanent changes to their education systems or generated a more-flexible common digital pedagogical model.

One of the challenges of future research is to map the path to a flexible, long-lasting pedagogical renovation. To master that challenge, we need not only organisational flexibility (flexibility in space and time), but also pedagogical/educational flexibility (flexibility in regard to teaching and learning). In other words, for a flexible learning model, we must answer the questions of when and where to learn (space and time), how to learn (way of learning/teaching), what to learn (contents) and with whom to learn (network and community) (Collis & Margaryan, 2007; Willems, 2011).

The subject is undeniably one of political importance, as demonstrated by the development of specific plans like the European Commission’s Digital Education Action Plan (2021–2027). Once political institutions put digital education on their agenda, terms like “online learning” and “blended learning” acquire shades of political meaning (Hodges et al., 2020). The research shows that during the COVID-19 pandemic the roll-out of a digital model depended on institutions’ own private policies, resulting in patchy application and uneven results (Hodges et al., 2020). There was no common basic model.

The implementation of a flexible digital pedagogy model will turn up variables that depend on political decisions, like the amount of teacher time to invest, the influence on passing requirement levels and institutional policies about assessment. This opens up an interesting line for future research based on examining the influence of European, national and institutional political variables on the implementation of a stable, consistent digital pedagogical model at universities.

The main limitation of this research is that it was based entirely on the Web of Science database. It did not include publications from the SCOPUS database, which would have furnished more data and would probably have afforded access to other publications, resulting in a more-thorough study. It was decided to use WoS only for three reasons. The first is that using both databases would have yielded repeated results. Furthermore, while SCOPUS could have offered a greater quantity of search results, those results would have been less selective, because SCOPUS is more inclusive than it is selective. The Web of Science is more selective and therefore offers better publications. This is fundamental in topics on which there is a large number of publications. The second reason is that most of the journals indexed in WoS are also indexed in SCOPUS, but the reverse is not true, because SCOPUS is less selective. The third reason is the wealth of databases WoS includes. WoS is the database most organisations would prefer to see their publications indexed in, and it is also the leading database for university promotion in most countries.

The conclusion is that this is a field that has come a long way but remains of great scientific interest today and is still developing (Croxall & Koh, 2013), a field whose object of study has changed time and time again. The field has moved on from studying educational technology and/or teaching with technology (inter alia, Koehler & Mishra, 2009) to exploring new methodologies and ways of teaching in today’s society (inter alia, Greenhow et al., 2021). The conclusion is that, while the medium is important, it remains simply another component of the process. The focus of analysis therefore ought to be shifted from medium to methodology. The important thing is not how we use media (Prestridge, 2012), but how to develop new, creative pedagogies in connection with technologies (Morris & Stommel, 2013).

## Data Availability

The datasets generated during and/or analysed during the current study are available from the corresponding author on reasonable request.
